# The Effects of ICT-Based Interventions on Physical Mobility of Older Adults: A Systematic Literature Review and Meta-Analysis

**DOI:** 10.1155/2023/5779711

**Published:** 2023-11-10

**Authors:** Hyori Kim, Gahye Kim, Yeonghun Kim, Jiyeon Ha

**Affiliations:** ^1^College of Nursing, Seoul National University, Seoul 03080, Republic of Korea; ^2^Robotics Lab, Hyundai Motor Company, Uiwang 16082, Republic of Korea; ^3^College of Nursing, Research Institute of Nursing Science, Ajou University, Suwon 16499, Republic of Korea

## Abstract

Systematic literature review and meta-analysis were conducted to integrate and analyze intervention studies dealing with the effects of information and communications technology- (ICT-) based interventions on the physical mobility of older adults in the community. The PubMed/MEDLINE, Embase, CINAHL, and Cochrane CENTRAL databases were searched for studies published from January 2000 to December 2022. We used the Risk of Bias 2 (RoB 2) tool to evaluate the quality of the randomized controlled studies in the systematic review. The meta-analysis was performed using a random-effects model. The model was used to calculate the standardized mean difference (SMD) and 95% confidence interval (CI) for both effect measures. *I*^2^ tests were used to measure the presence of heterogeneity. Thirty-seven randomized controlled trials were included (2,419 intervention participants), of which 23 were included in the meta-analysis. ICT interventions significantly improved Timed Up and Go (TUG) as a marker of physical mobility variable in older adults (SMD = −0.33, 95% CI: −0.57 to −0.10, *p*=0.005, *I*^2^ = 74.7%). A sensitivity analysis was performed on subgroups, and interventions were found to be effective in improving TUG in the exergame group (SMD = −0.40, 95% CI: −0.72 to −0.08, *p* < 0.001, *I*^2^ = 75.0%) and in the exergame with virtual reality (VR) group (SMD = −0.33, 95% CI: −1.01 to 0.35, *p* < 0.001, *I*^2^ = 91.0%) but both groups showed high heterogeneity. A meta-analysis was also performed on Short Physical Performance Battery (SPPB) but statistically significant results were not found (SMD = −0.19, 95% CI: −0.61 to 0.23, *p*=0.375, *I*^2^ = 87.7%). For the Berg Balance Scale (BBS), the post-intervention scores were significantly better than baseline (SMD = 1.52, 95% CI: 0.48 to 2.57, *p*=0.004, *I*^2^ = 93.5%). However, the number of studies included in the meta-analysis was small and heterogeneity was high, so follow-up studies are needed. This study confirmed that exergames, telecommunication, e-health, information applications, and robots were used as effective ICT-based interventions for improving the physical mobility of older adults. It is necessary to develop and apply more diverse ICT-based interventions that will prevent impairments of mobility and encourage older adults to live more independently, with a higher quality of life, based on extensive research on ICT-based interventions.

## 1. Introduction

The prolonged life expectancy and rapidly growing worldwide population of older adults have brought age-related physical, cognitive, and psychosocial health issues into the societal spotlight [1]. Older adults experience declining physical function (e.g., reduced muscle strength), impaired sensory function (e.g., vision and hearing), and decreased mobility caused by multiple factors, including reduced social activities after retirement [2]. In particular, maintaining mobility is an important goal for older adults to maintain independence and quality of life [3]. In older adults, reduced physical mobility is likely to have negative impacts on their life, including an increased likelihood of falling and hospitalization [4, 5], as well as placing them at higher risk for depression, social isolation, and loneliness [6, 7]. It has been established that senior citizens capable of standing for extended periods or traveling to various locations tend to have a lower risk of death [8], be more independent, and have a better quality of life [3].

The term “mobility” has various meanings depending on the context in which it is used [9]. Mobility, usually understood as a component of overall function, is defined as the ability to move or be moved easily and freely [10, 11]. Mobility is also used in a broader meaning, encompassing not only ambulation but also to participation in daily life or leisure activities, exercise, and using a variety of public transport modes [12, 13]. Given the multifaceted nature of mobility, methods to measure it are highly diverse. For example, physical activity, physical performance, muscle mass and strength, and balance and gait performance have been used to assess the level of mobility [3, 14]. Among several assessment tools, the Timed Up and Go (TUG) test, Short Physical Performance Battery (SPPB), and Berg Balance Scale (BBS) have frequently been used to evaluate older people's mobility [9]. In this review, mobility was defined as “physical mobility,” focusing on a person's physical ability to change his or her location or position or move from one place to another by walking and basic ambulation.

Recent advances in information and communications technology (ICT) have allowed the healthcare and medical sector to utilize the benefits of ICT in many ways, with impacts including reduced medical expenses, improved administrative tasks, maintaining patients' medical history, and reduced traditional paperwork [15]. A report released by Statista, a global statistics portal service, estimated that the global digital healthcare market in 2018 was worth USD 84.9 billion and was expected to grow to USD 504.4 billion by 2025 [16]. The use of telemedicine rapidly increased during the COVID-19 pandemic [17], and the COVID-19 pandemic has also resulted in enhancing digital acceptance among older adults [18]. Following this trend, ICT has been increasingly incorporated in various interventions for older adults to help with their daily routines and reduce healthcare costs [19]. According to a systematic literature review on ICT-related interventions for seniors, interventions using computers and the Internet, robotics, telemedicine, virtual reality, video games, and sensor technology have proven to be effective in lowering fall risk and social isolation, improving quality and satisfaction of life, increasing gait speed, and reducing depression [20]. Studies on ICT-based interventions to improve physical mobility in older adults include a pilot study that used an interactive smartphone application to boost physical activity in older adults [21] and a study on gait performance during wearable robot-assisted gait in older adults [22]. Another systematic literature review reported that exergame technology and interactive interventions contributed to higher mobility and enhanced balance in older adults [23, 24]. A more comprehensive analysis is needed to understand the effects of other types of ICT-based interventions on enhancing physical mobility among older adults, as more ICT-based interventions will be performed in the future.

Various types of ICT-based interventions are performed to promote physical mobility, which has positive effects on older adults' quality of life [25]. This study presents a systematic literature review and meta-analysis of studies on ICT-based interventions to promote mobility among older adults to provide a comprehensive and objective conclusion on the topic. This study will help understand the impacts of ICT-based interventions on improvements in physical mobility in the older population, given the trend for technological advancements, and can provide a foundation to promote successful aging through improving physical mobility and the quality of life in the older population.

## 2. Methods

### 2.1. Research Question

Systematic literature review and meta-analysis were conducted to verify the effects of ICT-related interventions on the physical mobility of older adults. This study was conducted according to the systematic literature review guidelines of Preferred Reporting Items for Systematic Reviews and Meta-analyses (PRISMA) [26].

Detailed data selection criteria were established as described below using PICO-SD, the key question strategy recommended in guidelines on systematic literature reviews [26]. The participants were community-dwelling older adults aged 65 and above, without physical limitations. Those with severe cognitive impairments were excluded. The interventions analyzed in this study utilized ICT modalities (e.g., the Internet, wireless networks, cell phones, and other equipment and technologies). Interventions using robotics, telemedicine, sensor technology, video games, smartphones, mobile applications, and medication-dispensing devices were included in this study [20, 27]. This study only included studies with control groups. The control groups for comparisons in this study were older adults (aged 65 and older) who did not receive interventions or those who received usual-care interventions for ethical purposes. The outcomes included measured variables of senior physical mobility. Physical mobility for the purpose of this study denoted the physical ability to move from one place to another (i.e., physical performance and physical activity). In this review, variables measuring physical mobility were classified into five categories: physical activity, physical performance, muscle mass and strength, and balance and gait performance [3, 14]. A meta-analysis was conducted on physical performance and balance, since these were the categories with sufficient studies to enable a meta-analysis [28].  Table 1 shows the measurement variables according to the classification of physical mobility used in this study. The study type was limited to randomized controlled trials (RCTs) only. Studies conducted among adults aged 65 and under or older adults residing in facilities including nursing homes, interventions not utilizing ICT, interventions that used ICT simply as a tool for contacting participants, and interventions with the main purpose of treating or rehabilitating a particular disease were excluded.

### 2.2. Search Strategy

Three researchers who had experience in meta-analyses and literature searches conducted the literature search for this study after receiving IRB approval (IRB No. KYU-2020-145-01), and its protocol has been registered in PROSPERO (No. CRD42021225483).

The search formula was created with a combination of terms representing the older population aged 65 and older (P) and ICT-based interventions (I). Four databases (PubMed/MEDLINE, Embase, CINAHL, and Cochrane CENTRAL) were selected based on the COSI model suggested by the National Library of Medicine (NLM). Relevant full publications and conference abstracts were identified by electronic searching of the four online databases using both text words and exploded Medical Subject Heading (MeSH) terms: (aged) AND (locomotion OR exercise OR Physical Functional Performance OR Walking Speed OR Muscle strength OR Postural Balance OR Mobility Limitation) AND (telemedicine OR information technology OR Information Science OR Robotics OR Video games OR Cell Phone OR Smartphone OR Mobile Applications). The results were limited to RCTs published in English between January 2000 and December 2022. In addition to the MeSH terms, text search terms were entered in the search. The detailed search formula is outlined in the Supplementary Material (Supplementary Appendix S1).

### 2.3. Data Extraction

The items of the data extraction form for systematic literature review were decided by consensus among the three researchers. The data analysis form included the author, published year, country of the study, place of the study, characteristics of inclusion/exclusion criteria, age and gender of participants, ICT intervention type, devices used, whether the study analyzed an individual or group intervention, the duration and frequency of the intervention, the intervention provider, the duration of follow-up, effect variables, and devices used for outcome measurements. If there were inconsistencies in the results among researchers, final decisions were made after reviewing and discussing the original studies.

### 2.4. Quality Assessment

The 37 selected studies were evaluated using the revised Cochrane risk-of-bias tool for randomized trials (RoB 2) for RCT studies developed by the Cochrane Bias Method Group [29]. The RoB 2 tool consists of 22 questions in five areas including randomization process, intended interventions, missing outcome data, measurement of the outcome, and reported results. The choices for answering each question were “yes,” “probably yes,” “probably no,” “no,” and “no information.” Each researcher decided whether the risk of bias was “low risk,” “some concerns,” or “high risk” and reevaluated the literature for questions where they had disagreements. The researchers reached a conclusion after sharing and discussing each other's evaluation records for these questions.

### 2.5. Statistical Analysis

For studies that were suitable for meta-analysis, the effect size and homogeneity of the ICT interventions were calculated using R version 4.2.1. A meta-analysis was conducted when four or more studies reported data on the same outcome variable [30]. Therefore, a meta-analysis was conducted on the TUG, SPPB, and BBS, which are commonly used to measure physical mobility [30]. The standardized mean difference (SMD) was used to quantify the effect size of outcome variables reported with different measurement tools or units, and mean difference (MD) was used when the measurement tools and units were the same. For a crossover study [31], since data at each starting point and end of follow-up were presented, each time point was regarded as a separate study and the standardized mean difference (SMD) was obtained and analyzed. In addition, for multiarm studies [32–34], the groups were combined and then analyzed [35]. A random-effects model was used under the hypothesis that each study would have different participants, intervention methods, and research environment. Heterogeneity was estimated using the forest plot, and statistical hypothesis testing was conducted using the *I*^2^ index to quantify the dispersion among the studies. An *I*^2^ value of higher than 75% means considerable heterogeneity, 25% < *I*^2^ ≤ 75% indicates moderate heterogeneity, and an *I*^2^ value of 25% or less means low heterogeneity [30]. If the outcome variables were measured twice or more, the value measured immediately after the intervention was adopted, considering that the results may be distorted with time, and the statistical significance of the effect size was evaluated using 95% confidence interval (CI) and a 5% level of significance. The MD between two groups was considered insignificant if the 95% CI included 0, while it was considered significant if the 95% CI did not include zero. The interpretation of the effect size was based on Cohen's standardized mean difference, where 0.20 ≤ *d* ≤ 0.50 denotes a small effect, 0.50 ≤ *d* ≤ 0.80 indicates a medium effect, and *d* ≥ 0.80 denotes a large effect [36]. The funnel plot, Begg and Mazumdar's rank correlation test, and Egger's linear regression test methods were used to evaluate publication bias.

## 3. Results

**Figure 1:**
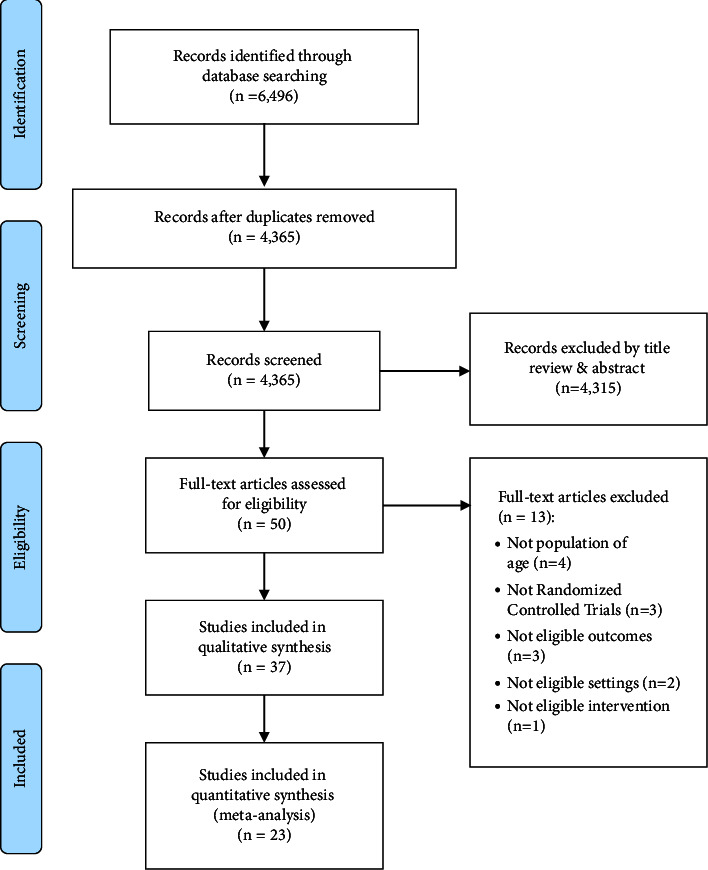
Flow diagram of the publication search process.

This study reviewed the existing literature to identify the effects of ICT-based interventions on the physical mobility of older adults. The online database search yielded a total of 6,496 studies, including 2,493 from PubMed/MEDLINE, 1,719 from CINAHL, 2,154 from Embase, and 130 from the Cochrane CENTRAL. The number of overlapping studies from the first search was 2,131. Of the 4,365 studies, 50 were selected after applying the inclusion and exclusion criteria upon reviewing the titles and abstracts. Thirty-seven studies were finally selected for analysis, removing four studies that did not match the age criteria, three non-RCT studies, five studies that did not have eligible outcomes and research environment criteria, and one study that did not fit in terms of the intervention. 23 studies with measurements of the same variables were finally selected for the meta-analysis (Figure 1).

### 3.1. Systematic Review

#### 3.1.1. Summary of the Included Studies

The characteristics of the studies finally selected for the systematic literature review are presented in Table 2. Four (10.8%) studies were conducted before 2010, while 33 (89.2%) studies were published after 2010. Of particular note, 24 studies were published after 2020. The studies were carried out in 22 countries distributed across five continents, including the US (eight studies) [38–40, 51, 62–64, 67], South America (one study) [61], Europe (10 studies) [1, 41, 43, 44, 48, 53, 54, 57, 60, 65], Middle East (four studies) [45, 49, 50, 68], Asia (nine studies) [31–33, 47, 52, 55, 56, 58, 66], Australia/Oceania (four studies) [34, 37, 42, 61], and Africa (one study) [59]. The participants were both male and female in 35 studies, while one study had female participants [46], and one study had male participants [57]. Eight studies identified only the gender of the total participants and did not specify the gender ratio of the intervention group and control group [34, 40, 45, 54, 56, 57, 59, 61].

In the 37 selected studies, the number of participants in the intervention groups included in the analysis was 2,282 and the number of participants in the control groups was 1,996. The sample size ranged from 9 to 585 participants. Seven studies had a sample size of 15 or less in the intervention group [1, 31, 39, 41, 54, 56, 61], and one of these had a randomized crossover study design [31]. One study only reported total participants [40]. The mean age of the participants (intervention group) ranged from 68.2 to 85.1 years old, respectively.

#### 3.1.2. Types of the ICT-Based Interventions

**Figure 2:**
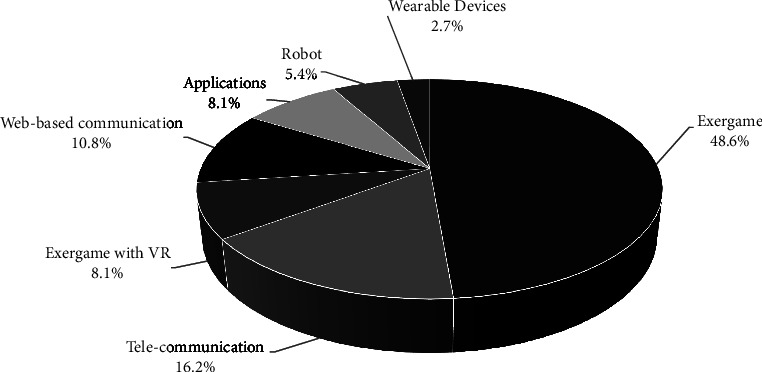
Distribution of types of interventions on physical mobility of older adults.

Details of ICT-based interventions are presented in Tables 2 and 3. The types of ICT interventions included exergames in a majority of the studies [1, 31, 39, 44–50, 52, 54, 58, 59, 61–63, 68] as well as exergames with virtual reality (VR) [33, 34, 41], telecommunications [40, 51, 53, 57, 64, 67], web-based communication [37, 42, 43, 65], applications [38, 60, 66], robots [32, 56], and wearable devices [55] (Figure 2).

In the types of devices used in the ICT interventions (counting overlapping devices), personal computers [1, 62, 63] and game platforms (Nintendo Wii, Xbox, and StepMania) [31, 39, 44–50, 52, 54, 58, 59, 61, 68] were used in exergame interventions, and VR goggles, Oculus, HTC, and game platforms (e.g., Xbox) were used for exergames with VR [33, 34, 41].

Telecommunications interventions mainly used telephones [40, 51, 57, 64], although smartphones [53] and video conferencing units [53, 67] were used, too. In application-based interventions, tablets and smartphones were used, and accelerometers, personal computers, smartphones, and tablets were used in web-based communication interventions [38, 60, 66]. A balance exercise assist robot and an exoskeletal hip-assist robot were used in robot interventions [32, 56], and wearable motion sensors were used in a study of wearable devices [55].

The contents of the interventions were exercise in 19 studies [31–33, 39, 41, 45–50, 52–56, 62, 67, 68], exercise and cognitive functioning training in seven studies [1, 34, 43, 44, 58, 61, 63], exercise motivation-boosting programs in five studies [37, 40, 51, 57, 65], motivation for exercise and health advice (including nutritional advice) in five studies [38, 42, 60, 64, 66], and cognitive functional training interventions in one study [62].

Individual interventions were performed in 30 studies [1, 31, 32, 34, 37–39, 41–43, 45, 47–52, 54–58, 60, 61, 63–68], group interventions in four studies [33, 44, 46, 59], and a mixture of individual and group interventions in three studies [40, 53, 62].

The duration of the interventions ranged from 2 weeks to 48 weeks, and the weekly frequency of interventions (per week) ranged from 0.5 to 5 and 6 to 80 minutes per session. The highest proportion of interventions in 21 studies was self-performed using ICT, while others were performed by researchers in seven studies, or with third-party professionals including trainers, counselors, instructors, supervisors, or physiotherapists in 10 studies.

#### 3.1.3. The Characteristics of the Older Adults' Physical Mobility Measurement Variables

The 37 studies used different variables to measure the physical mobility of older adults (Tables 1 and 3). Physical activity (Houston Physical Activity Scale (HPAS), Community Healthy Activities Model Program for Seniors (CHAMPS) questionnaire, leisure physical activity, Short Questionnaire to Assess Health Enhancing Physical Activity (SQUASH), etc.) was measured in nine studies (24.3%), gait performance (single-task walking (m/s), Digiwalkers, 10 m walk, gait speed, etc.) was measured in 15 studies (40.5%), physical performance (SPPB, TUG, Functional Reach Test (FRT), 6-minute walk test (6MWT), etc.) was measured in 26 studies (70.3%), balance (BBS, 30 s chair rises test, 5 times sit-to-stand, etc.) was measured in 18 studies (48.6%), and muscle mass and strength (30 s chair-rises test, 5 times sit-to-stand, knee extensor strength, skeletal muscle mass, etc.) were measured in 15 studies (40.5%).

### 3.2. Effects by Type of ICT Intervention

#### 3.2.1. Exergames

By intervention type, 18 studies used exergames, and the intervention group had 611 participants (range, 4 to 186) including 353 females (except for those that did not report gender), compared to 589 (range, 5 to 186) in the control group, including 342 females. The sample size ranged from 9 to 372 participants in total [1, 31, 39, 44–50, 52, 54, 58, 59, 61–63, 68]. The mean age was 74.01 in the intervention group and 74.21 in the control group.

In terms of the contents of interventions, 12 studies provided exercise interventions [31, 39, 45–50, 52, 54, 59, 68], five studies provided exercise and cognitive function training [1, 44, 58, 61, 63], and one study provided only cognitive function training [62]. The interventions were self-conducted by the participants in seven studies [1, 47, 49, 50, 54, 61, 62] and by a trainer, researcher, or expert in 11 studies [31, 39, 44–46, 48, 52, 58, 59, 63, 68]. The mean frequency and duration of the interventions were 45.88 minutes/session and 2.42 sessions/week for 9.17 weeks. Among the mobility variables measured in this study, TUG was most frequently reported to be significant in 13 studies [31, 39, 45–49, 52, 58, 59, 61, 62, 68], while two studies reported no effectiveness results for TUG [39, 45].

Each study suggested that it was effective to measure physical balance and function as a variable, with examples including gait speed [1, 44, 63], SPPB [1, 44, 63], BBS [31, 39, 49, 68], FRT [39, 47, 59], the 30 s chair-stand test [1, 48], center of pressure (COP) [45, 48, 50], 6 minute walk test (m) [52, 59], and 5 times sit-to-stand [46, 61]. Each study reported that the above variables were effective, but the gait speed [63] and FRT [47] results did not show significant differences, and BBS and FRT showed no significant differences in one study [39].

#### 3.2.2. Exergames with VR

Three studies used exergames with VR, and the intervention group had 101 participants (range, 13 to 45) including 10 females (except for those that did not report gender), compared to 41 (range, 11 to 15) in the control group, including 10 females. The sample size ranged from 24 to 60 participants in total [33, 34, 41]. The mean age was 76.29 in the intervention group and 77.90 in the control group.

In terms of the contents of interventions, two studies provided exercise interventions [33, 41], and one study provided exercise and cognitive function training [34]. The interventions were provided by a trainer/researcher [33] or physiotherapists [34, 41]. The mean frequency and duration of the interventions were 25.33 minutes/session and 3 sessions/week for 7 weeks.

Among the mobility variables measured in this study, TUG was most frequently reported to be significant in all three studies. It was confirmed that there was a significant improvement compared to the pre-test in the 10 m walk test and single-leg stance test, and significant changes were also reported in variables such as the Performance Oriented Mobility Assessment (POMA) [34, 41] and muscle strength [33]. It was also reported that the VR intervention group exhibited improved physical mobility compared to the control group [33, 34].

#### 3.2.3. Telecommunication

Six studies used telecommunication technologies for their interventions [40, 51, 53, 57, 64, 67]. The number of participants in the intervention and control groups was 354 (range, 28 to 234) including 231 females and 317 (range, 13 to 160) including 210 females, respectively (with the exclusion of one study because it did not report the number of participants) [40]. The sample size ranged from 41 to 351 participants in total, and the mean age was 73.79 in the intervention group and 73.87 in the control group.

In terms of the contents of interventions, two studies provided exercise interventions [53, 67], three studies provided motivation for exercise [40, 51, 57], and one study provided motivation for exercise and nutritional advice [64]. In one study [64], the intervention was self-conducted by the participants, while in other studies, the interventions were provided by researchers [40, 51] or counselors [57], or were self-conducted by the participants with an educational-professional physical trainer [53]. The interventions were provided for an average of 44.67 minutes, 1.79 times/week, for 14.67 weeks.

These studies demonstrated effectiveness in terms of improving single leg stance (SLS), medial-lateral foot center of pressure (ML-COP), and TUG scores [67] and increasing physical activity [51, 64, 67]. Venditti et al. [64] reported that SPPB, gait speed, and 5-chair-stand components demonstrated effectiveness compared to the pre-test, but there were no significant differences between groups. Furthermore, Langeared et al. [53] reported that a videoconferencing-based exercise intervention was partially effective in strengthening muscles (knee and lower limb) but was not as effective as a face-to-face exercise intervention in improving knee flexion isometric strength.

#### 3.2.4. Web-Based Communication

Three studies performed interventions by providing information using web-based communication technologies [42, 43, 65], and one study used web-based communication with wearable devices [37]. The number of participants in the intervention group was 662 (range, 60 to 254) including 407 females, the control group had 662 participants (range, 60 to 264) including 415 females, and the sample size ranged from 120 to 503 participants in total. The mean age was 73.10 in the intervention group and 73.55 in the control group.

Two studies provided motivation for exercise [37, 65], one study motivated exercise and provided health advice [42], and other study provided exercise, cognitive training, and nutritional advice [43]. The intervention was self-provided via the app or the web page [42, 43, 65], and one study was provided by an expert [37]. The interventions lasted for 11.75 weeks.

Delbaere et al. [42] demonstrated effectiveness in terms of improving standing balance and functional mobility (such as TUG, the 5 times sit-to-stand test, 10 m walk, and SPPB) compared to the pre-test, but there were no significant differences between groups. Alley et al. [37] reported that the intervention improved moderate to vigorous physical activity (MVPA) outcomes in comparison with a control group.

#### 3.2.5. Applications

Two studies used applications [38, 66] and one study used an application with wearable devices [60]. The number of participants in the intervention group was 259 (range, 80 to 150) including 164 females, while the control group had 662 participants (range, 60 to 264) including 415 females, and the sample size ranged from 157 to 263 participants in total. The mean age was 69.94 in the intervention group and 70.80 in the control group.

In terms of the contents of interventions, all studies provided motivation for exercise and presented health or nutritional advice. The interventions were self-conducted by the participants in two studies [38, 66] and by a medical doctor for 24 weeks in another study [60].

Regarding outcome variables, inconsistent results were reported for gait step—namely, Bickmore et al. [38] reported that the intervention group participants walked significantly more than control participants, but Recio-Rodríguez et al. [60] reported that there was no significant difference between the groups. Wang et al. [66] reported that the intervention improved skeletal muscle mass, but that it was particularly effective in the group where exercise and nutritional counseling were also provided.

#### 3.2.6. Robots

Robotic technology was used in two intervention studies, and the intervention group had 29 participants (range, 14 to 15), compared to 26 (13 participants each) in the control group. The sample size ranged from 27 to 58 participants in total [32, 56]. One study reported only total participants' sex ratio and age (20 females among 29 participants; mean age, 73) [56], and another study's intervention group had 21 females (mean age, 74.2) compared to 7 females (mean age, 76.4) in the control group [32]. All studies provided exercise interventions where participants performed the interventions themselves with the help of researchers. The mean frequency and duration of the interventions were 40 minutes/session and 2.5 sessions/week for 8 weeks. The outcome variables showed inconsistent results; Ozaki et al. [56] reported that the TUG, FRT, muscle strength (abduction, extension, adduction, and flexion—hip and knee), and gait speeds exhibited a significant effect compared to the control group. Lee et al. [32] reported that the SPPB, TUG, FRT, and muscle strength (abduction, extension, adduction, flexion—hip and knee, and trunk) demonstrated effectiveness compared to the pre-test, but there were no significant between-group differences.

#### 3.2.7. Wearable Devices

Wearable devices were used in one intervention study [55], where participants performed the intervention with assistance from researchers. The intervention group had 36 participants including 16 females (mean age, 69.3), and the control group had 34 participants including 16 females (mean age, 68.8).

The exercise interventions were provided for 60 minutes/session and 3 times/week for 12 weeks. The study reported that the Chair-Stand-30 (CS-30) scores and other physical function outcomes (TUG and CS-30) improved in both the intervention and control groups, but there was no statistically significant difference between the groups.

### 3.3. Quality Evaluation of the Literature

**Figure 3:**
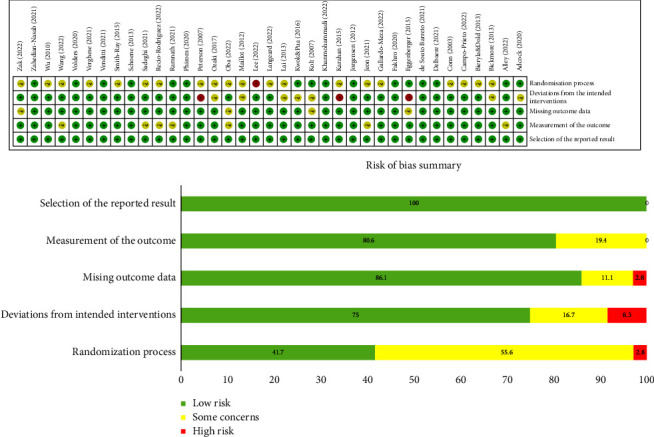
Risk of bias summary in included studies. The 37 publications were evaluated for their risk of bias using the tool to implement RoB 2 provided by the Cochrane Group. The evaluation of missing outcome data estimated that two studies had some concerns because the dropout rate of the initial participants was 5% or more, and all others were evaluated as low risk. There was deemed to be a low risk of bias for the measurement of the outcome and the reported results.

The 37 publications were evaluated for their risk of bias using Microsoft Excel tool in accordance with RoB 2, provided by the Cochrane Group. In the first area of the randomization process, 16 studies (42.1%) were evaluated as low risk because they followed the randomization process relatively well with a detailed outlining of the randomization process and hiding the assignment order, while 21 studies (55.3%) were evaluated as having some concerns because they did not detail the process of randomization (Figure 3). One study (2.6%) was randomized but not blinded to participants and classified as high risk [32].

In terms of the intended interventions, 28 studies (76.3%) were evaluated as low risk because they included statistical information on participants who were excluded from the intended intervention or dropped out but had a small impact on the outcomes. Three studies (7.9%) were evaluated as high risk because 5% or more of the participants were excluded from the analysis. Seven studies (15.8%) were evaluated as having some concerns about carrying out a modified intention-to-treat protocol because they included all participants except for those with missing outcomes or it was unclear whether the participants or researchers were aware of the randomization.

The evaluation of missing outcome data estimated that three studies (7.9%) had some concerns because the dropout rate of the initial participants was 5% or more, and all others were evaluated as low risk. For measurements of the outcomes, seven studies (18.4%) had some concerns because a double-blind study was not conducted; thus, the outcome assessors may have been aware of the interventions that the study participants received. There was deemed to be a low risk of bias for the reported results (Figure 3).

### 3.4. Meta-Analysis Outcomes by Intervention

#### 3.4.1. Timed Up and Go (TUG)

**Figure 4:**
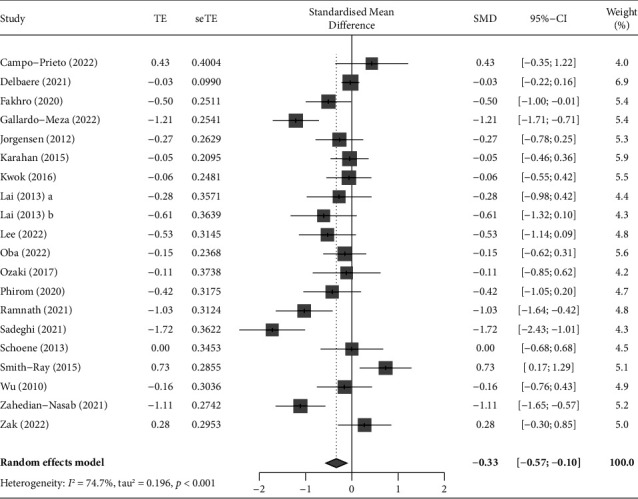
Forest plot displaying the results of a meta-analysis of the outcome (TUG) of ICT-based intervention in community-dwelling older adults. The standardized mean difference (SMD) was used to quantify the effect size of outcome variables reported with different measurement tools or units, and the pooled results significantly showed that ICT-based interventions could improve TUG of older adults significantly (SMD = −0.33, 95% CI: −0.57 to −0.10, *p*=0.005).

Of the 37 studies, 20 used TUG as an outcome variable [31–34, 41, 42, 45–49, 52, 55, 56, 58, 59, 61, 62, 67, 68]. A meta-analysis was performed of 19 studies with a verified effect size [31–34, 41, 42, 45, 46, 48, 49, 52, 55, 56, 58, 59, 61, 62, 67, 68], unifying the effect size of each outcome using Hedges' g value and distribution. One study was excluded from the meta-analysis because the post-test data of the control group were not presented [47]. The pooled results significantly showed that ICT-based interventions could improve TUG of community-dwelling older adults significantly (SMD = −0.33, 95% CI: −0.57 to −0.10, *p*=0.005, Figure 4), but the analysis showed that the 20 studies were heterogeneous (*I*^2^ = 74.7%, *Q* = 75.08, *p* < 0.001).

**Figure 5:**
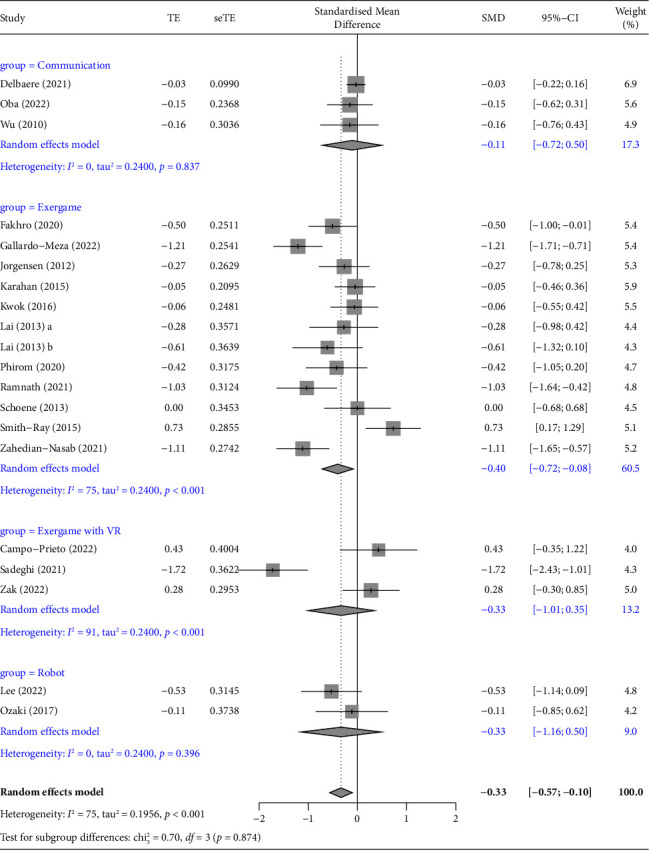
Forest plot displaying the results of a subgroup analysis of the outcome (TUG) of ICT-based intervention in older adults.

A subgroup analysis was performed because the original meta-analysis was performed with different kinds of ICT interventions in a single analysis, which could lead to bias in interpreting the outcomes. In the subgroup analysis, in 12 exergame studies [31, 45, 46, 48, 49, 52, 58, 59, 61, 62, 68] and three exergame with VR studies [33, 34, 41], TUG significantly decreased in the post-test compared to the pre-test, proving that the interventions had effects. However, both groups showed high heterogeneity (exergame *I*^2^ = 74.7%, VR with exergame *I*^2^ = 91.1%) (Figure 5). To assess the impact of an individual study on the pooled estimates, a sensitivity analysis was conducted by excluding one study at a time. For TUG, the sensitivity analyses yielded similar results, indicating that no individual study influenced the TUG (Supplementary Figure 1).

#### 3.4.2. Short Physical Performance Battery (SPPB)

**Figure 6:**
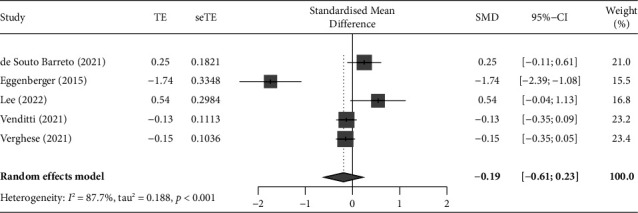
Forest plot displaying the results of a meta-analysis of the outcome (SPPB) of ICT-based intervention in community-dwelling older adults. The standardized mean difference (SMD) was used to quantify the effect size of outcome variables reported with different measurement tools or units. The pooled results were not significant; there is insufficient evidence that ICT-based interventions could improve SPPB in community-dwelling older adults (SMD = −0.19, 95% CI: −0.61 to 0.23, *p*=0.375).

Of the 37 studies, six used SPPB as an outcome variable. A meta-analysis was performed of five studies with a verified effect size [32, 43, 44, 63, 64], unifying the effect size of each outcome using Hedges' g value and distribution. One study was excluded from the meta-analysis because standard deviations were not presented [1]. The pooled results were not significant; thus, there is insufficient evidence that ICT-based interventions could improve SPPB in community-dwelling older adults (SMD = −0.19, 95% CI: −0.61 to 0.23, *p*=0.375, Figure 6), and the analysis showed that the five studies were heterogeneous (*I*^2^ = 87.7%, *Q* = 32.43, *p* < 0.001).

#### 3.4.3. Berg Balance Scale (BBS)

**Figure 7:**
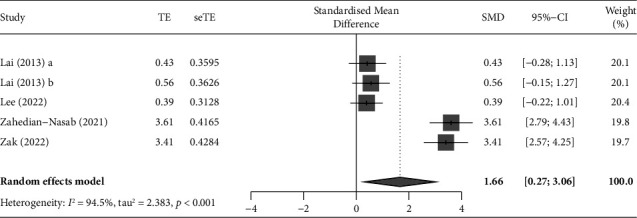
Forest plot displaying the results of a meta-analysis of the outcome (BBS) of ICT-based intervention in community-dwelling older adults. The standardized mean difference (SMD) was used to quantify the effect size of outcome variables reported with different measurement tools or units, and the pooled results showed that ICT-based interventions could significantly improve BBS in community-dwelling older adults (SMD = 1.52, 95% CI: 0.48 to 2.57, *p*=0.004).

Of the 37 studies, six used BBS as an outcome variable. A meta-analysis was performed of five studies with a verified effect size [31, 32, 34, 49, 68], unifying the effect size of each outcome using Hedges' g value and distribution. One study was excluded from the meta-analysis because standard deviations were not presented [39]. The pooled results showed that ICT-based interventions could significantly improve BBS in community-dwelling older adults (SMD = 1.52, 95% CI: 0.48 to 2.57, *p*=0.004, Figure 7), but the analysis showed that the studies were heterogeneous (*I*^2^ = 93.5%, *Q* = 76.43, *p* < 0.001).

### 3.5. Publication Bias Outcomes

**Figure 8:**
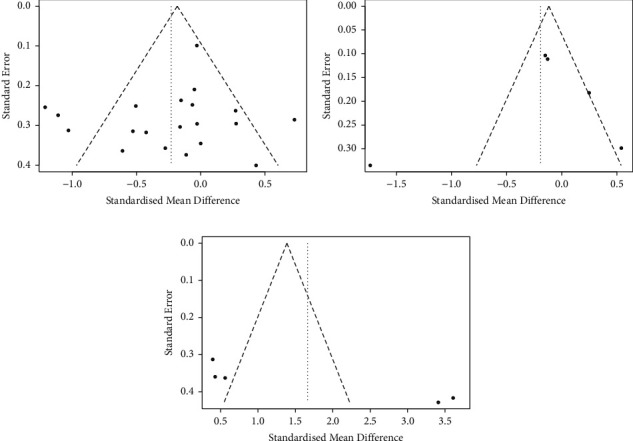
Funnel plots with TUG (a), SPPB (b), and BBS (c) as the outcome variable of ICT-based intervention for the community-dwelling older adults. (a) Funnel plots with Timed Up and Go (TUG). (b) Funnel plots with Short Physical Performance Battery (SPPB). (c) Funnel plots with Berg Balance Scale (BBS).

We ran Egger's test and Begg's test to assess whether there was any potential publication bias for studies with TUG as the outcome variable (Egger linear regression: *t* = −0.846, *p*=0.408; Begg rank correlation: *Z* = −0.649, *p*=0.516), Egger's test, Begg's test, and funnel plots did not identify any publication bias. The studies with SPPB (Egger linear regression: *t* = −0.350, *p*=0.749; Begg rank correlation: *Z* = 0.490, *p*=0.624) did not show any publication bias in Egger's test and Begg's test, but the funnel plot appeared asymmetrically, so publication bias could not be ruled out. However, studies with BBS (Egger linear regression: *t* = 3.376, *p*=0.043; Begg rank correlation: *Z* = 1.960, *p*=0.050) may have been subjected to potential publication bias (Figures 8(a)–8(c)).

## 4. Discussion

The purpose of this study was to verify the effects of ICT-based interventions on the physical mobility of older adults through a systematic literature review and meta-analysis of RCTs. In total, 37 studies were selected for the systematic literature review. A meta-analysis was performed of the 23 studies that reported TUG, SPPB, or BBS outcome variables to identify the effect size.

Twenty-four of the 37 studies were published after 2020, reflecting the recent trend for more publications as ICT-based interventions gained attention during the coronavirus disease 2019 (COVID-19) pandemic. Among the various types of interventions, exergames were used in 18 studies, accounting for almost half (47.4%; reaching 55.3% if including exergame using VR), followed by telecommunications, one of the most traditional types of ICT (six studies, 15.8%). This is in line with the results of previous systematic literature review studies suggesting that exergames are commonly used as an effective intervention for promoting physical activities or mobility among older adults [24, 69, 70]. Exergames readily triggered interest and motivation among participants, bringing higher compliance and persistence than traditional exercise interventions, and are considered a cost-effective intervention to encourage physical activity [39, 48, 54]. Research has suggested that contactless interventions based on the web or telecommunications had markedly higher compliance than in-person interventions because providing in-person interventions at a certain venue made it inconvenient for the participants to be there at a certain time, even if the physical distance was minimal [38, 40, 67]. This is a meaningful finding given the current circumstances with rising demand for the development and application of contactless healthcare interventions throughout the COVID-19 pandemic. It shows that applying ICT in various healthcare interventions can be effective for increasing compliance by triggering interest and facilitating convenience among participants. In particular, during the COVID-19 pandemic, ICT-related health intervention services were effective for supplementing face-to-face intervention services in situations where it was difficult to deliver health services face-to-face [71, 72]. However, it is difficult to comply with regulations (intensity, frequency, posture, etc.) for ICT services compared to face-to-face services, so related guidelines are needed [53]. Therefore, in order to prepare for these issues, prior training on posture when providing ICT services, periodic feedback from experts, and measures for safety issues would be essential [71].

This study also found that telecommunications-based interventions, as a type of classic ICT, were cost-effective in boosting physical mobility among community-dwelling older adults. This conclusion is supported by another study that found telephone counseling to be effective in encouraging moderate physical activity [51, 57] and a study reporting that using both telephone and mail for exercise counseling was more effective in promoting exercise among participants than simply using mail only [40]. Interventions that provided exercise information using applications or websites were also effective in promoting physical mobility among older adults [38, 65], as were interventions using robots [56]. A systematic literature review of these studies confirmed that most ICT-based interventions, including training sessions using exergames and robots, as well as information-based interventions such as web-based interventions, applications, and telecommunications, were effective in improving mobility among older adults.

Interventions that used computer games and gaming devices for cognitive training exclusively or in combination with exercise interventions were effective for improving physical mobility [44, 61, 62]. This result is consistent with the outcomes of existing systematic literature reviews proving that individuals' physical mobility can improve as a result of utilizing ICT-based virtual reality for learning and processing diverse information in the central nervous system such as visual images, exercise planning, and motivation [52, 61], and that general cognitive training can improve mobility among older adults, especially in the context of higher-order executive function (such as walking while talking) [73]. The study published by Smith-Ray et al. [62] also confirmed that cognitive training using ICT improved the range of field of view, driving ability, and confidence, implying their effectiveness for mobility. However, considering the potential safety concerns such as fall risk and injury when applying ICT-based interventions for older adults in the community, it is important to make sure that the interventions are safely performed with the help of researchers or trained medical staff [39, 48, 52, 56, 61]. In addition, an introductory session on how to safely use ICT devices should be provided prior to ICT-based interventions so that participants can better understand ICT-related interventions and apply them in a safe manner and ensure higher compliance and lower dropout rates [31, 38, 49, 52, 54].

In particular, with the recent development of VR technology, VR exergames have been introduced. Compared to regular exergames, VR allows greater immersion in the situation, thereby increasing interest [33, 34, 41]. Compared to exergames alone, physical mobility was further increased by exergame interventions with VR, most likely because VR-based exergames require much greater sensory integration and processing [33]. This fact can be used as a motivational strategy for participation in mobility promotion interventions for the elderly.

Variables for measuring the physical mobility of older adults included measures of primary outcomes, such as physical activity (e.g., International Physical Activity Questionnaire (IPAQ), MVPA, etc.), physical performance (e.g. SPPB, TUG test, etc.), muscle mass and strength (e.g. 30s chair stand test, skeletal muscle mass, muscle function, etc.), balance (e.g. BBS, standing balance (s), etc.), and gait performance (e.g., single-task walking (m/s), step count, 6MWT, etc.). In the meta-analysis, it was difficult to analyze the integrated effect size across the studies due to the inconsistent mobility measurement variables. 21 out of the 37 selected studies reported TUG as an outcome variable, and a meta-analysis was performed on the 20 studies in which the effect size could be confirmed. ICT interventions were found to be effective in improving TUG as a physical mobility measurement variable. A sensitivity analysis was performed for each subgroup, and the exergame and exergame with VR groups showed high heterogeneity, but TUG decreased from the pre-test to the post-test, proving that it was effective.

In addition, a meta-analysis was performed on SPPB and BBS. For BBS, the post-intervention effect was significant. However, the number of studies used in the meta-analysis is small and heterogeneity is high, so follow-up studies are needed. Although TUG, SPPB, and BBS are widely used clinical assessment tools to evaluate balance and walking ability among the older adults, the concept of mobility in the elderly is a complex concept [3], so a tool that can evaluate these factors together is needed.

This study is meaningful in that it verified the effects of ICT-based interventions on the physical mobility of community-dwelling older adults through a systematic and objective integration of individual studies. Most of the studies included in the analysis had a low risk of bias, and only RCT studies were included to ensure credibility. However, this study had limitations in that it only included studies published in the English language. In addition, this study included interventions with a small sample size (15 or fewer participants) [1, 31, 39, 41, 54, 56, 61] in the meta-analysis. This requires attention due to the possibility of overestimating the effect size. Therefore, to compensate for this issue, Hedges' g value was used in the meta-analysis. If the number of studies included in a meta-analysis is less than 10, the statistical test may not detect heterogeneity among the studies. For the outcome variables of SPPB and BBS, the number of included studies was less than ten; thus, there may have been heterogeneity that was not found in this study. Furthermore, for TUG, the limitations were supplemented by identifying heterogeneity-related factors through subgroup analysis. In this study, a meta-analysis was performed with only papers published in academic journals, which was motivated by the need to include high-quality articles on this topic. However, it has been pointed out that a meta-analysis can produce more reliable results when studies published in academic journals and unpublished studies are included in the analysis at a similar ratio [74]. Thus, a follow-up meta-analysis incorporating unpublished studies at an appropriate ratio is recommended.

Most of the studies excluded from this study dealt with ICT interventions provided to older adults, focusing on rehabilitation from certain conditions, including dementia, stroke, and Parkinson's disease, as well as patients who had undergone musculoskeletal surgery, implying the need for further research on developing and expanding ICT-based interventions effective for promoting mobility among healthy community-dwelling older adults. Additionally, this study included interventions conducted in 22 countries distributed across five continents. Each country has different levels of digitalization and different levels of informatization, and these characteristics may affect the themes or effectiveness of interventions due to the level of development of the information society [75]. Therefore, the findings of this study should be interpreted carefully. We also suggest analyzing whether differences exist in the type and effect of ICT intervention depending on the level of digitalization.

## 5. Conclusion

This study systematically reviewed research on the effects of ICT-based interventions on physical mobility among community-dwelling older adults and conducted a meta-analysis to determine the effect size of TUG, SPPB, and BBS, a variable for measuring physical mobility in older adults. The outcomes demonstrated that ICT interventions using exergames, e-health, information applications, and robots were effective in enhancing senior physical mobility, as well as telecommunication interventions (as the most traditional ICT intervention). Moreover, ICT interventions were effective in enhancing physical mobility. In the future, more diverse ICT-based interventions should be developed and provided to older adults in the community to prevent impairments of mobility and cognitive function and to encourage older adults to live more independently, with a higher quality of life, based on extensive research on ICT-based interventions.

## Figures and Tables

**Table 1:** Measurement variables according to the classification of physical mobility.

Categories	Name of assessment
Physical activity	Moderate to vigorous physical activity time by accelerometer, steps/day, % of days with valid step counts, Baecke physical activity scale, rating of perceived exertion, HPAS, self-reported questionnaire (QAPPA), continuous values of metabolic equivalent task/week (MET-min/week), IPAQ, accelerometer (%, steps per minute), leisure physical activity (min/week), SF-36 (physical functioning and role functioning/physical scale), physiological profile assessment (PPA), CHAMP, MVPA (accelerometer for 7 days), SQUASH
Physical performance	Extended balance test of SPPB, FRT, TUG, FTSTS, 6MWT, Functional Reach Test (cm), XMSS (Xavix measured step system) stepping test, CS-30
Muscle mass and strength	Senior fitness test (30 s chair-rises test, 2 min stepping test), 5 times sit-to-stand, MVC, RFD, 30 s chair-stand test, KES, knee extension strength, knee flexion strength, lower limb power, chair stand (muscle strength for lower body), executive function tests (trail making test B, digit symbol substitution test, letter fluency test), skeletal muscle mass, muscle function
Balance	Standing balance (s), COP, one-leg static balance, dynamic balance 10 m test (s), POMA, COP-VM (center of pressure velocity moment), BBS, UST, 8-foot up and go dynamic/static balance (COP), single-leg stance test on firm and foam surfaces, tandem stance test, ML-COP, SLS, FAB
Gait performance	Single-task walking (m/s), Digiwalkers, maximum anteroposterior leaning range, coordinated lean score, 10 m walk, stepping reaction time, gait speed, step count, step counts from accelerometers, 6-minute walk test

HPAS = Houston Physical Activity Scale; QAPPA = quantization autotuner for precision programmable accelerators; IPAQ = International Physical Activity Questionnaire; SF-36 = 36-item short form survey; CHAMPS = Community Healthy Activities Model Program for Seniors questionnaire; MVPA = moderate to vigorous physical activities; SQUASH = Short Questionnaire to Assess Health Enhancing Physical Activity; SPPB = Short Physical Performance Battery; FRT = Functional Reach Test; TUG = Timed Up and Go; FTSTS = five times sit-to-stand test; 6-MWT = 6 minute walk test; CS-30 = 30-sec chair stand test; MVC = maximum voluntary contraction; RFD = rate of force development; KES = knee extensor strength; COP = center of pressure; POMA = Performance Oriented Mobility Assessment; COP-VM = center of pressure velocity moment; BBS = Berg Balance Scale; UST = unipedal stance test; SLS = single leg stance; ML-COP = medial-lateral foot center of pressure; FAB = Fullerton Advanced Balance Scale.

**Table 2:** Summary of the included studies evaluating the effects of ICT-based interventions on physical mobility of older adults.

Author	Country	Analyzed sample size		M : W		Age (M ± SD)		Intervention		Outcomes related to physical mobility
		IG	CG	IG	CG	IG	CG	Type of ICT	Type of interventions	Major outcome variables
Adcock et al. (2020) [1]	Switzerland	15	16	5 : 10	10 : 6	77.0 ± 6.4	70.9 ± 5.0	Exergame	Exercise and cognitive training	(1) Gait performance: single-task walking (m/s)
										(2) Physical performance: extended balance test of SPPB
										(3) Muscle mass and strength: senior fitness test (30 s chair-rises test, 2 min stepping test)

Alley et al. (2022) [37]	Australia	78	69	18 : 60	15 : 54	69.9 ± 4.1	68.8 ± 3.9	Web-based communication and wearable devices	Motivation for exercise and Fitbit	(1) Physical activity: moderate to vigorous physical activity (MVPA) time by accelerometer
		96	—	19 : 77	—	69.1 ± 4.9	—		Motivation for exercise	

Bickmore et al. (2013) [38]	USA	132	131	43 : 89	59 : 72	71.7 ± 5.6	70.8 ± 5.2	Applications	Motivation for exercise and health advice	(1) Physical activity: steps/day, % of days with valid step counts

Bieryla and Dold (2013) [39]	USA	4	5	NR	NR	82.5 ± 1.6	80.5 ± 7.8	Exergame	Exercise	(1) Balance: BBS (Berg Balance Scale), FAB (Fullerton Advanced Balance Scale)
										(2) Physical performance: FRT (functional reach), TUG (Timed Up and Go)

Conn et al. (2003) [40]	USA	NR	NR	NR	NR	74.0 ± 6.4	75.8 ± 7.4	Telecommunication	Motivation for exercise with telephone calls and mailed materials	(1) Gait performance: Digiwalkers
		NR	—	NR	—	75.1 ± 5.9	—		Motivation for exercise	(2) Physical activity: Baecke physical activity scale, rating of perceived exertion, HPAS (Houston Physical Activity Scale)
		NR	—	NR	—	75.2 ± 7.2	—		Telephone calls and mailed materials	

Campo-Prieto et al. (2022) [41]	Spain	13	11	3 : 10	1 : 10	85.1 ± 8.5	84.8 ± 8.1	Exergame with VR	Exercise	(1) Balance: Tinetti test (POMA)
										(2) Physical performance: TUG, FTSTS (five times sit-to-stand test)

Delbaere et al. (2021) [42]	Australia	254	249	77 : 177	87 : 162	77.1 ± 5.5	77.7 ± 5.5	Web-based communication	Motivation for exercise and health advice	(1) Balance: standing balance (s)
										(2) Gait performance: 10 m walk; stepping reaction time
										(3) Physical performance: TUG, SPPB
										(4) Muscle mass and strength: 5 times sit-to-stand, maximum AP (anteroposterior) leaning range, coordinated lean score

de Souto Barreto et al. (2021) [43]	France	60	60	29 : 31	22 : 38	75.2 ± 5.7	73.2 ± 5.3	Web-based communication	Exercise, cognitive training, and nutritional advice	(1) Gait performance: gait speed, step count, step counts from the accelerometer
										(2) Physical activity: self-reported questionnaire (QAPPA), continuous values of metabolic equivalent task/week (MET-min/week)
										(3) Physical performance: SPPB

Eggenberger et al. (2015) [44]	Switzerland	24	25	8 : 16	9 : 16	78.5 ± 5.1	80.8 ± 4.7	Exergame	Exercise	(1) Gait performance: velocity, step length, step length variability, step time, step time variability
										(2) Physical performance: SPPB, 6-MWT

Fakhro et al. (2020) [45]	Lebanon	33	31	NR	NR	72.2 ± 5.2	76.4 ± 8.7	Exergame	Exercise	(1) Balance: CoP
										(2) Physical performance: TUG

Gallardo-Meza et al. (2022) [46]	Chile	35	37	0 : 35	0 : 37	69.2 ± 3.7	68.1 ± 3.3	Exergame	Exercise	(1) Balance: one-leg static balance
										(2) Physical performance: TUG
										(3) Muscle mass and strength: 5-repetition sit-to stand

Jeon et al. (2021) [47]	South Korea	22	19	11 : 11	9 : 10	71.3 ± 4.2	72.8 ± 5.2	Exergame	Exercise	(1) Balance: POMA
										(2) Physical performance: TUG, FRT

Jorgensen et al. (2012) [48]	Denmark	28	30	9 : 19	9 : 21	75.9 ± 5.7	73.7 ± 6.1	Exergame	Exercise	(1) Balance: CoP-VM (center of pressure velocity moment)
										(2) Physical performance: TUG
										(3) Muscle mass and strength: MVC (maximum voluntary contraction), RFD (rate of force development), 30 s chair stand test

Karahan et al. (2015) [49]	Turkey	48	42	27 : 21	24 : 18	71.3 ± 6.1	71.5 ± 4.7	Exergame	Exercise	(1) Physical performance: TUG
										(2) Balance: BBS

Khanmohammadi et al. (2022) [50]	Iran	28	29	13 : 16	13 : 15	71.8 ± 4.2	71.2 ± 5.8	Exergame	Exercise	(1) Balance: CoP-related parameters (mediolateral (ML) and anteroposterior (AP) displacement and velocity)

Kolt et al. (2007) [51]	USA	93	93	35 : 58	28 : 65	74.1 ± 6.2	74.3 ± 5.9	Telecommunication	Motivation for exercise	(1) Physical activity: leisure physical activity (min/wk), SF-36∼physical functioning, role physical

Kwok and Pua (2016) [52]	Singapore	40	40	4 : 36	8 : 32	70.5 ± 6.7	69.8 ± 7.5	Exergame	Exercise	(1) Gait performance: 6-minute walk test
										(2) Physical performance: TUG
										(3) Muscle mass and strength: KES (knee extensor strength)

Lai et al. (2013) [31]	Taiwan	15	15	7 : 8	6 : 9	70.6 ± 3.5	74.5 ± 4.7	Exergame	Exercise	(1) Balance: BBS, UST
										(2) Physical performance: XMSS (Xavix measured step system) stepping test, TUG

Langeard et al. (2022) [53]	France	13	13	4 : 9	5 : 8	73.0 ± 4.0	74.0 ± 4.0	Telecommunication	Exercise (videoconference)	(1) Muscle mass and strength: knee extension strength, knee flexion strength, lower limb power
		15	—	6 : 9	—	72.0 ± 4.0	—		Exercise (face to face)	

Lee et al. (2022) [32]	South Korea	15	13	8 : 7	6 : 7	75.2 ± 3.4	76.4 ± 5.0	Robot	Exercise with robot (walking)	(1) Balance: BBS
		15	—	9 : 6	—	72.7 ± 3.1	—		Exercise with robot (stair ascent)	(2) Physical performance: SPPB, TUG, FRT
		15	—	7 : 8	—	74.7 ± 5.1	—		Exercise with robot (treadmill walking)	(3) Gait performance: 10 m walk test for self-selected velocity

Maillot et al. (2012) [54]	France	15	15	NR	NR	73.5 ± 4.1	73.5 ± 3.0	Exergame	Exercise	(1) Balance: 8-foot up and go
										(2) Gait performance: 6 min walk (cardiorespiratory fitness)
										(3) Muscle mass and strength: chair stand (muscle strength for lower body)

Oba et al. (2022) [55]	Japan	36	34	19 : 17	18 : 16	69.3 ± 3.9	68.8 ± 3.1	Wearable devices	Exercise	(1) Physical performance: CS-30, TUG

Ozaki et al. (2017) [56]	Japan	14	13	NR	NR	NR	NR	Robot	Exercise	(1) Balance: dynamic/static balance (COP)
										(2) Gait performance: gait speed
										(3) Physical performance: FRT (Functional Reach Test), TUG
										(4) Muscle mass and strength

Peterson et al. (2007) [57]	UK	26	29	26 : 0	29 : 0	73.0 ± 4.0	74.0 ± 4.0	Telecommunication	Motivation for exercise (in frail)	(1) Gait performance: 6 min walk (feet), gait velocity (m/s)
		13	13	13 : 0	13 : 0	72.0 ± 4.0	—		Motivation for exercise (not frail)	(2) Muscle mass and strength: chair stands (n)

Phirom et al. (2020) [58]	Thailand	20	20	3 : 17	4 : 16	70.2 ± 4.2	69.4 ± 3.4	Exergame	Exercise and cognitive training	(1) Physical activity: physiological profile assessment (PPA)
										(2) Physical performance: Timed Up and Go test (TUG)

Ramnath et al. (2021) [59]	South Africa	23	22	NR	NR	70.8 ± 4.5	74.1 ± 5.8	Exergame	Exercise	(1) Balance: dynamic balance 10 m test (s)
										(2) Gait performance: 6 min walk test (m)
										(3) Physical performance: TUG, Functional Reach Test (cm)

Recio-Rodríguez et al. (2022) [60]	Spain	81	79	31 : 50	31 : 48	69.9 ± 3.6	71.7 ± 6.2	Applications and wearable devices	Motivation for exercise and nutritional advice	(1) Physical activity: IPAQ, accelerometer (%, steps per minute)

Sadeghi et al. (2021) [33]	Malaysia	14	15	14 : 0	15 : 0	70.4 ± 4.3	72.2 ± 7.2	Exergame with VR	Balance training	(1) Balance: single-leg stance test on firm and foam surfaces, tandem stance test
		15	—	15 : 0	—	74.1 ± 7.0	—		VR exercise	(2) Gait performance: 10 m walk test
		14	—	14 : 0	—	70.5 ± 5.1	—		Balance training and VR exercise	(3) Physical performance: TUG
										(4) Muscle mass and strength

Schoene et al. (2013) [61]	Australia	15	17	NR	NR	77.5 ± 4.5	78.4 ± 4.5	Exergame	Exercise and cognitive training	(1) Physical performance: TUG
										(2) Muscle mass and strength: 5 times sit-to-stand

Smith-Ray et al. (2015) [62]	USA	27	24	6 : 21	6 : 18	82.7 ± 6.0	81.1 ± 6.8	Exergame	Training cognitive function with computer game	(1) Gait performance: 10 m walk
										(2) Physical performance: TUG

Verghese et al. (2021) [63]	USA	186	186	51 : 135	50 : 136	76.9 ± 5.7	77.1 ± 5.6	Exergame	Exercise and cognitive training	(1) Gait performance: walking speed
										(2) Physical performance: SPPB
										(3) Muscle mass and strength: executive function tests (trail making test B, digit symbol substitution test, letter fluency test)

Venditti et al. (2021) [64]	USA	162	160	36 : 126	39 : 121	71.3 ± 4.4	71.0 ± 4.2	Telecommunication	Motivation for exercise and nutritional advice	(1) Gait performance: gait speed (m/s)
										(2) Physical activity; CHAMP
										(3) Physical performance: SPPB
										(4) Muscle mass and strength: 5-chair rise (s)

Volders et al. (2020) [65]	Netherlands	260	325	138 : 122	164 : 161	74.2 ± 6.6	74.5 ± 6.2	Web-based communication	Motivation for exercise	(1) Physical activity: MVPA (accelerometer for 7 days), SQUASH

Wang et al. (2022) [66]	China	50	51	8 : 42	7 : 44	70.2 ± 4.3	69.9 ± 3.3	Applications	Motivation for exercise and nutritional advice	(1) Muscle mass and strength: skeletal muscle mass, muscle function
		50	—	10 : 40	—	69.7 ± 3.6	—		Motivation for exercise	
		51	—	9 : 41	—	68.2 ± 3.9	—		Nutritional advice	

Wu et al. (2010) [67]	USA	22	20	3 : 19	4 : 16	76.1 ± 7.9	74.1 ± 6.9	Telecommunication	Exercise with custom made video conferencing unit	(1) Balance: ML-COP, SLS
		22	—	3 : 19	—	75.9 ± 6.3	—		Exercise with DVD	(2) Physical performance: TUG

Zahedian-Nasab et al. (2021) [68]	Iran	30	30	22 : 8	22 : 8	69.7 ± 7.7	72.0 ± 7.8	Exergame	Exercise	(1) Balance: BBS
										(2) Physical performance: TUG

Zak et al. (2022) [34]	New Zealand	15	15	NR	NR	76.7 ± 1.5	76.7 ± 1.6	Exergame with VR	Exercise	(1) Balance: BBS, POMA, single-leg stance test
		15	—	NR	—	78.1 ± 3.7	—		Exercise and cognitive training	(2) Gait performance: 10 m walk
		15	—	NR	—	79.1 ± 3.6	—		Exercise and cognitive training with Oculus	(3) Physical performance: TUG

M : W = men : women; IG = intervention group; CG = control group; SPPB = Short Physical Performance Battery; BBS = Berg Balance Scale; FAB = Fullerton Advanced Balance Scale; FRT = Functional Reach Test; TUG = Timed Up and Go; HPAS = Houston Physical Activity Scale; POMA = Performance Oriented Mobility Assessment; FTSTS = five times sit-to-stand test; QAPPA = quantization autotuner for precision programmable accelerators; 6-MWT = 6 minute walk test, COP = center of pressure; IPAQ = International Physical Activity Questionnaire; CoP-VM = center of pressure velocity moment; MVC = maximum voluntary contraction; RFD = rate of force development; SF-36 = 36-item short form survey; KES (knee extensor strength); UST = unipedal stance test; CS-30 = 30-sec chair stand test; CHAMPS = Community Healthy Activities Model Program for Seniors questionnaire; MVPA = moderate to vigorous physical activity; SQUASH = Short Questionnaire to Assess Health Enhancing Physical Activity; ML-COP = medial-lateral foot center of pressure; SLS = single leg stance.

**Table 3:** Details of ICT-based interventions on physical mobility of older adults.

Author	Intervention								
	Type of ICT	Type of intervention	ICT device used	Individual/group	Provider	Duration (weeks)	Frequency (per week)	Time (min)	Control group
Adcock et al. (2020) [1]	Exergame	Exercise and cognitive training	Inertial measurement units (IMUs) providing both accelerometer and gyroscope assessments	Individual	Self	16–18	3	30–40	Usual care
Alley et al. (2022) [37]	Web-based communication and wearable devices	Motivation for exercise	Web-based massage using if-then algorithms, Fitbit	Individual	Expert	12	NR	NR	Usual care
Bickmore et al. (2013) [38]	Applications	Motivation for exercise and health advice	Home tablet computers, kiosk computers	Individual	Self	48	NR	NR	Usual care
Bieryla and **D**old (2013) [39]	Exergame	Exercise	Nintendo's Wii Fit game (balance board)	Individual	Self (with a supervisor)	3	3	30	Usual care
Conn et al. (2003) [40]	Telecommunication	Motivation for exercise	Telephone (and mail)	Individual and group	Researcher	12	3	30	Usual care
Campo-Prieto et al. (2022) [41]	Exergame with VR	Exercise	HTC Vive ProTM	Individual	Physiotherapists	10	3	6	Usual care
Delbaere et al. (2021) [42]	Web-based communication	Motivation for exercise and health advice	A tablet computer	Individual	Self	9	NR	NR	Usual care
de Souto Barreto et al. (2021) [43]	Web-based communication	Exercise, cognitive training and nutritional advice	A tablet (model: HP x2 210 G2-10.1), a commercial wrist-worn accelerometer (model: Fitbit flex 2), smartphone	Individual	Self	12	2	NR	Usual care
Eggenberger et al. (2015) [44]	Exergame	Exercise and cognitive training	GAITRite electronic walkway system (CIR systems, Havertown, PA, USA) with the Platinum Version 4.0 software	Groups of 5-6	Researcher	26	1	60	Usual care
Fakhro et al. (2020) [45]	Exergame	Exercise	Wii balance board	Individual	Researcher	4	1	40	No intervention
Gallardo-Meza et al. (2022) [46]	Exergame	Exercise	Wii balance board	Group	Self, but senior undergraduate physiotherapists to ensure safety and maintenance	4	2	40	Usual care
Jeon et al. (2021) [47]	Exergame	Exercise	Wii Fit	Individual	Self	2	5	NR	Lower intensity exercise program than intervention group
Jorgensen et al. (2012) [48]	Exergame	Exercise	Nintendo's Wii Fit game (balance board)	Individual	Self with a trained physiotherapist	10	2	60∼80	Usual care
Karahan et al. (2015) [49]	Exergame	Exercise	Xbox KinectTM device	Individual	Self	6	5	30	Usual care
Khanmohammadi et al. (2022) [50]	Exergame	Exercise	Wii Fit	Individual	Self	5	3	60	Motor-cognitive dual task training with variable-priority instruction
Kolt et al. (2007) [51]	Telecommunication	Motivation for exercise	Telephone and mail	Individual	Researcher	12	1	10.2 ± 5.3∼16.5 ± 6.9	Usual care
Kwok and Pua (2016) [52]	Exergame	Exercise	Nintendo's Wii Fit game	Individual	Self with a trained physiotherapist	12	1	60	Usual care
Lai et al. (2013) [31]	Exergame	Exercise	Computer-based cognitive training program	Individual	Trainer	6	3	30	No intervention
Langeard et al. (2022) [53]	Telecommunication	Exercise	Smartphone (video conference program)	Individual and group	Self, education-professional physical trainer	16	2	60	No intervention
Lee et al. (2022) [32]	Robot	Exercise	Exoskeletal hip-assist robot	Individual	Self with researcher	4	3	40	No intervention
Maillot et al. (2012) [54]	Exergame	Exercise	Nintendo Wii video game console and Nintendo Wii balance board	Individual (paired with another)	Self	14	1	60	No intervention
Oba et al. (2022) [55]	Wearable devices	Exercise	Wearable motion sensors (named “Moff-Trai”)	Individual	Researcher	12	3	60	Usual care with the light-load exercise program of watching a video on a health maintenance
Ozaki et al. (2017) [56]	Robot	Exercise	BEAR (balance exercise assist robot) (Toyota Motor Corporation, Aichi, Japan)	Individual	Self (but with assistant)	12	2	NR	Usual care
Peterson et al. (2007) [57]	Telecommunication	Motivation for exercise	Telephone	Individual	Counselor	24	0.5∼1	NR	Usual care
Phirom et al. (2020) [58]	Exergame	Exercise and cognitive training	A Microsoft® Xbox 360 Kinect sensor V2, LED projector, and laptop computer	Individual	Researcher	12	3	60	Usual care
Ramnath et al. (2021) [59]	Exergame	Exercise	The Xbox Kinect sports video gaming software	Group	An exercise physiologist who acted as a “spotter,” an exercise physiologist	12	1	30	Exercise program consisting of a combination of standing and seated exercises
Recio-Rodríguez et al. (2022) [60]	Applications and wearable devices	Motivation for exercise and nutritional advice	Smartphone, smart watch	Individual	MD	12	NR	10	Usual care
Sadeghi et al. (2021) [33]	Exergame with VR	Balance training and exercise	Xbox (the sport Xbox Kinect game package)	Group	Trainer	8	3	40	No intervention
Schoene et al. (2013) [61]	Exergame	Exercise and cognitive training	The open-source DDR game StepMania	Individual	Self	8	2∼3	20	Usual care
Smith-Ray et al. (2015) [62]	Exergame	Training cognitive function with computer game	Computer	Individual and group	Self	10	3	60	No intervention
Verghese et al. (2021) [63]	Exergame	Exercise and cognitive training	Personal computer (CogniFit software)	Individual	Self	8	3	50	Usual care with interactive computer-based health education classes and a low complexity, nonprogressive program
Venditti et al. (2021) [64]	Telecommunication	Motivation for exercise and nutritional advice	Telephone	Individual	Masters level, licensed registered dietitians with geriatric nutrition expertise	12	1	60	Usual care with newsletter maintenance (mail/e-mail)
Volders et al. (2020) [65]	Web-based communication	Motivation for exercise	Internet such as e-mail (if they have registered an e-mail address)	Individual	Self	14	NR	NR	Usual care
Wang et al. (2022) [66]	Applications	Motivation for exercise and nutritional advice	Application (APP), including dietary or exercise assessments, feedback, and recommendations for improvement	Individual	Self	12	NA	NA	No intervention
Wu et al. (2010) [67]	Telecommunication	Exercise	Custom-made video conferencing unit, the DocBox (a Polycom VSX 7000b and 3 large-screen TV monitors)	Individual	Instructor (not researcher)	12	3	60	Usual care, not connected to the instructor
Zahedian-Nasab et al. (2021) [68]	Exergame	Exercise	Xbox Kinect	Individual	Researcher	6	1	30–60	Usual care
Zak et al. (2022) [34]	Exergame with VR	Exercise and cognitive training	Carl Zeiss VR One goggles, Oculus	Individual	Physiotherapists	3	3	30	N/A

## Data Availability

The data supporting this study are from previously reported studies and datasets, which have been cited. The processed data are available from the corresponding author upon request.

## Supplementary Materials

Supplementary Materials Supplementary Appendix S1: the strategy of search terms. Supplementary Figure 1: sensitivity analysis verifying the robustness of the outcome (Timed Up and Go test measurement) of ICT-based intervention in community-dwelling older adults by removing one study at a time.
