# Supplementary material for: The Effects of ICT-Based Interventions on Physical Mobility of Older Adults: A Systematic Literature Review and Meta-Analysis
Source: Int J Clin Pract. 2023 Nov 10;2023:5779711. doi: 10.1155/2023/5779711 (PMC10656205; doi:10.1155/2023/5779711)
Supplement: Supplementary Materials — Supplementary Appendix S1: the strategy of search terms. Supplementary Figure 1: sensitivity analysis verifying the robustness of the outcome (Timed Up and Go test measurement) of ICT-based intervention in community-dwelling older adults by removing one study at a time. [file 5779711.f1.zip › Supplementary Appendix S1.doc]

**Supplementary Appendix S1.** The strategy of search terms

| **Search item** | **Context** | | | | |
| --- | --- | --- | --- | --- | --- |
|  | **PICOs** | **MeSH** | **EMtree** | **CINAHL subject headings** | **Text (on title/abstract)** |
| Keywords used | Participants /Population | aged | aged | aged | aged OR elder* OR older* OR senior |
|  | Outcome | locomotion OR exercise OR Physical Functional Performance OR Walking Speed OR Muscle strength OR Postural Balance OR Mobility Limitation | locomotion OR exercise OR movement (physiology) OR physical activity OR physical performance OR walking speed OR walking difficulty OR walk test OR body equilibrium or muscle strength | Movement OR Mobility (Iowa NOC) OR Human Activities OR postureOR muscle strength OR Activity and Exercise Enhancement (Iowa NIC) | mobility OR physical activity OR physical performance OR exercise OR walking OR gait OR locomotion OR locomotor OR locomotory OR balance OR postural OR posture OR walking speed OR muscle strength |
|  | Interventions | telemedicine OR information technology OR Information Science OR Robotics OR Video games OR Cell Phone OR Smartphone OR Mobilie Applications | information and communication technology OR technology OR information processing OR information science OR information technology device OR robotics OR video game OR mobile phone OR mobile application OR mass communication | Information Science OR Technology OR Technology Managemnet (Iowa NIC) | "information and communication technology" OR ICT OR technolog* OR telemedicine OR "mobile health" OR "mHealth" OR "mobile phone" OR E-mail OR smartphone* OR "text message" OR computer OR game* OR robot* OR Web-based |
|  | Exclusion | nothing | | | |
|  | Study Design | RCT only | | | |
| Databases | PubMed/MEDLINE, Embase, CINAHL, and The Cochrane library | | | | |
| Time filter | 2000.01.01. ~ 2022. 12. 31. | | | | |
| Language | English | | | | |

PICO=Patient population, Intervention, Comparison, Outcomes; Iowa NOC=the Nursing Outcomes Classification developed by a research team at the University of Iowa; Iowa NIC=the Nursing Interventions Classification developed by a research team at the University of Iowa; RCT=Randomized Controlled Trials
