# Supplementary material for: The Effects of ICT-Based Interventions on Physical Mobility of Older Adults: A Systematic Literature Review and Meta-Analysis
Source: Int J Clin Pract. 2023 Nov 10;2023:5779711. doi: 10.1155/2023/5779711 (PMC10656205; doi:10.1155/2023/5779711)
Supplement: Supplementary Materials — Supplementary Appendix S1: the strategy of search terms. Supplementary Figure 1: sensitivity analysis verifying the robustness of the outcome (Timed Up and Go test measurement) of ICT-based intervention in community-dwelling older adults by removing one study at a time. [file 5779711.f1.zip › Supplementary Figure 1.doc]

**Supplementary Figure 1**. Sensitivity analysis verifying the robustness of the Outcome (Time Up and Go test measurement) of ICT-based intervention in community dwelling older adults by removing one study at a time.
